# Research on the Application of Silver Nanowire-Based Non-Magnetic Transparent Heating Films in SERF Magnetometers

**DOI:** 10.3390/s25010234

**Published:** 2025-01-03

**Authors:** Yi Ge, Yuhan Li, Yang Li, Xuejing Liu, Xiangmei Dong, Xiumin Gao

**Affiliations:** 1 School of Optical-Electrical and Computer Engineering, University of Shanghai for Science and Technology, Shanghai 200093, China; 223330867@st.usst.edu.cn (Y.G.); 2235062502@st.usst.edu.cn (Y.L.); liuxuejing@usst.edu.cn (X.L.); dxm77@usst.edu.cn (X.D.); gxm@usst.edu.cn (X.G.); 2 Qinhuangdao Hongyan Optoelectronics Technology Co., Ltd., Qinhuangdao 066000, China

**Keywords:** SERF atomic magnetometer, silver nanowires, magnetically transparent heating

## Abstract

We propose a non-magnetic transparent heating film based on silver nanowires (Ag-NWs) for application in spin-exchange relaxation-free (SERF) magnetic field measurement devices. To achieve ultra-high sensitivity in atomic magnetometers, the atoms within the alkali metal vapor cell must be maintained in a stable and uniform high-temperature environment. Ag-NWs, as a transparent conductive material with exceptional electrical conductivity, are well suited for this application. By employing high-frequency AC heating, we effectively minimize associated magnetic noise. The experimental results demonstrate that the proposed heating film, utilizing a surface heating method, can achieve temperatures exceeding 140 °C, which is sufficient to vaporize alkali metal atoms. The average magnetic flux coefficient of the heating film is 0.1143 nT/mA. Typically, as the current increases, a larger magnetic field is generated. When integrated with the heating system discussed in this paper, this characteristic can effectively mitigate low-frequency magnetic interference. In comparison with traditional flexible printed circuits (FPC), the Ag-NWs heating film exhibits a more uniform temperature distribution. This magnetically transparent heating film, leveraging Ag-NWs, enhances atomic magnetometry and presents opportunities for use in chip-level gyroscopes, atomic clocks, and various other atomic devices.

## 1. Introduction

With the advancement of atomic magnetic field measurement technologies, superconducting quantum interference devices (SQUIDs) and spin-exchange relaxation-free (SERF) magnetometers have gained widespread use in fields such as magnetoencephalography (MEG) [1,2,3,4,5] and magnetocardiography (MCG) [6,7,8,9,10] due to their non-destructive properties and exceptional sensitivity. Compared to SQUIDs, which require expensive and complex cryogenic systems, SERF atomic magnetometers offer advantages such as compact size, high sensitivity, and operation at ambient temperature [11,12]. Currently, the world’s most sensitive SERF atomic magnetometer achieves a sensitivity of 160 $aT/\sqrt{Hz}$, setting a new record in magnetic field measurement [13]. Nevertheless, a significant challenge hindering the sensitivity of SERF atomic magnetometers is the heating required for the alkali metal vapor cell [14,15]. To maintain sufficient atomic density, the alkali metal vapor cell must be heated to vaporize and stabilize the thermal stability of its internal atoms [16,17,18]. The uniformity and stability of this heating are crucial for preserving a high-temperature environment, directly impacting the uniformity of spin polarization of alkali metal atoms and consequently influencing the overall magnetometer sensitivity. Hence, optimizing the heating techniques and structure of alkali metal vapor cells to improve heating stability and uniformity is imperative. This improvement not only elevates magnetometer performance but also propels atomic field measurement technologies for applications in medical and scientific research, providing more precise and efficient tools for studying intricate phenomena such as neural activity and cardiac magnetic fields [19,20].

Presently, there are three commonly used heating methods: hot air flow heating [21,22], laser heating [23,24,25,26,27], and electric heating [28,29]. Hot air flow heating utilizes a blower to blow hot air into the alkali metal cell, ensuring heating without noise disturbance but causing significant airflow disturbance. Its bulky structure complicates miniaturization difficult and compromises temperature control precision and stability. Laser radiation of various wavelengths, including but not limited to infrared, can be used to heat the alkali metal cell, provided the wavelength is not resonant with the species involved, avoiding additional magnetic field introduction by utilizing non-resonant laser wavelengths. However, the instability of the incident heating laser source can cause interference, and its lower heating power and complex optical design limit its practical application in miniaturized atomic magnetometers [30].

In comparison, electric heating remains the mainstream choice due to its simpler design and easier controllability compared to the other two methods. Nonetheless, the inherent magnetic noise from heating currents impacts spin exchange relaxation of atoms, thereby diminishing magnetometer sensitivity [31,32]. Various methods to suppress magnetic field noise from electric heating have been proposed, such as intermittent electric heating [33] and high-frequency AC electric heating [34]. Additionally, different heating membrane structures have been explored, such as those made from Micro-Electro Mechanical Systems (MEMS) [35]. An innovative electric heating technology, referred to as the magnetic field elimination heater architecture [36,37], has recently emerged. This architecture effectively mitigates the magnetic field noise produced during the heating process by employing a configuration characterized by a 2N multiple magnetic moment alongside a reverse winding arrangement. Nevertheless, the complexity of this design, coupled with the prohibitively high costs associated with its manufacturing and debugging processes, renders it impractical for large-scale production. Currently, the most widely used heater in electric heating is the flexible printed circuit (FPC) heating film, which is adhered to the surface of the alkali metal cell oven with light transmission holes reserved to heat the internal chamber via oven heat transfer. This method often causes uneven internal chamber heating and condensation at the light transmission hole positions [38]. To address this issue, Gao et al. [39] proposed a new dual-layer cylindrical oven structure as an alternative to traditional square ovens to improve the temperature uniformity in alkali metal vapor cells. However, this approach is not conducive to miniaturization. Schwindt et al. [40] attempted to apply transparent heaters fabricated using MEMS processing techniques to chip-scale atomic magnetometers. However, this approach required bonding two parts together with epoxy resin, which is not conducive to mass production and makes it difficult to effectively eliminate current-induced magnetic noise. Liu et al. [41] employed glass as a substrate and coated it with indium tin oxide (ITO) to fabricate a transparent heating film. However, the adoption of ITO has been limited due to its high production costs and brittle characteristics, which can pose challenges in certain applications [42].

We propose a non-magnetic transparent heating film based on silver nanowires (Ag-NWs) aimed at replacing traditional FPC heating films. Ag-NWs exhibit low resistivity, high conductivity, and good transmittance in both the visible and near-infrared spectra; however, their stability may be compromised under high-temperature conditions [43,44,45]. Experimental results indicate that the Ag-NWs-based non-magnetic transparent heating film outperforms traditional FPC heating films in terms of heating uniformity and suppression of magnetic field noise. Furthermore, no significant degradation in stability was observed under elevated temperature conditions. This innovative Ag-NWs-based heating film therefore represents a promising advancement, poised not only for enhancing atomic magnetometer applications but also for optimizing the heating mechanisms of various other atomic devices.

## 2. Theoretical Analysis

Heat transfer is a complex physical process governed by the second law of thermodynamics, which dictates that heat spontaneously flows from a region of higher temperature to one of lower temperature when a temperature gradient exists. Heat transfer occurs through three primary mechanisms: conduction, radiation, and convection. The heating element converts electrical energy into thermal energy, which is then transferred through conduction to the surface of the alkali metal vapor cell, ensuring uniform heat distribution. Consequently, the outer walls of the vapor cell release the absorbed heat into the gas contained within, facilitating thermal equilibrium within the system. Concurrently, both the heating element and the vapor cell emit thermal radiation, contributing to the establishment of a dynamic thermal equilibrium within the system. The process of heat conduction at this stage can be described by Fourier’s law of heat conduction [46]:

(1) $$q=-\lambda (\vec{i}\frac{\partial T}{\partial x}+\vec{j}\frac{\partial T}{\partial y}+\vec{z}\frac{\partial T}{\partial z}),$$

where q represents the heat flux density, with units of W/m^2^; λ signifies thermal conductivity—a material’s capability to conduct heat, varying with factors like temperature and purity. Different materials have different conductivities, and even the same material’s conductivity changes with these factors. The negative sign in Equation (1) indicates that heat flows in the direction of decreasing temperature, opposing the temperature gradient.

In this study, the Ag-NWs’ transparent heating film heats the alkali metal vapor chamber via surface heating. To address surface heat sources, we consider their spatial distribution and impact on heat conduction. A simplified 2D heat transfer model is used, with the film thickness fixed. This allows us to focus on the volume heat generation intensity and its distribution within the film, avoiding the complexity of a 3D model. Assuming the heat source is distributed over a plane with a given area and intensity (heat per unit volume), the 2D heat conduction equation is expressed as:

(2) $$\frac{\partial T(x,y,t)}{\partial t}=\alpha (\frac{\partial^{2}T(x,y,t)}{\partial x^{2}}+\frac{\partial^{2}T(x,y,t)}{\partial y^{2}})+\frac{Q(x,y)}{\lambda },$$

where, T(x,y,t) denotes the temperature at position (x,y) as a function of time t. The parameter α represents the diffusion coefficient ($\alpha =\frac{\lambda }{\rho c_{p}}$), ρ is the density, and $c_{p}$ is the specific heat capacity. Q(x,y) represents the distribution of the heat source.

Considering the variation in heat source intensity with distance, and assuming the thermal energy at the center of the heat source follows a Gaussian distribution, substituting this distribution into the heat conduction equation gives:

(3) $$\frac{\partial T(x,y,t)}{\partial t}=\alpha (\frac{\partial^{2}T(x,y,t)}{\partial x^{2}}+\frac{\partial^{2}T(x,y,t)}{\partial y^{2}})+\frac{Q_{0}\exp(-\frac{{\left(x-x_{s}\right)}^{2}+{\left(y-y_{s}\right)}^{2}}{2\sigma^{2}})}{\lambda },$$

When the entire system reaches dynamic thermal equilibrium, the resulting thermal balance equation is:

(4) $$Q=\rho C_{p}\frac{\partial T(x,y,t)}{\partial t}+\rho C_{p}v\cdot \nabla T(x,y,t)+\nabla \cdot \left[-k\cdot \nabla T(x,y,t)\right],$$

where Q represents the total input heat to the system, ρ is the density of the object, v is the fluid velocity, and ∇ is the Nabla operator (also known as the Hamiltonian operator), which also has a vector form, representing temperature changes in different directions in space. In the experiment, the alkali metal chamber is securely integrated with the Ag-NWs’ transparent heating film and housed within an insulated apparatus. Within the framework of our system, it is assumed that the temperature of the system at its boundaries is equal to the ambient environmental temperature. Upon reaching dynamic equilibrium, conduction becomes the dominant mode of heat transfer, while thermal convection and radiation can be considered negligible.

## 3. Experimental Setup

**Figure 1:**
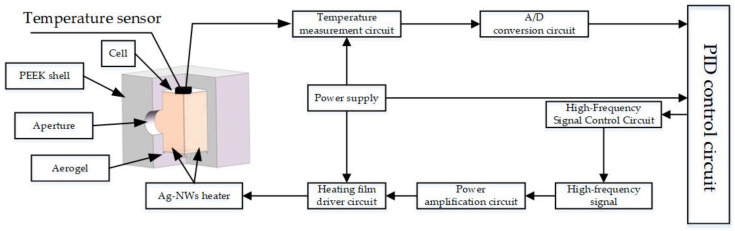
Non-magnetic electric heating structure.

The atomic magnetometer primarily comprises an alkali metal vapor cell, a pump laser, a probe laser, a magnetic shield, and a non-magnetic heating system. The non-magnetic heating system is responsible for heating the alkali metal vapor cell to the required operating temperature while ensuring that magnetic noise interference is minimized during the heating process. This study employs electrical heating to elevate the temperature of the alkali metal vapor cell. As depicted in Figure 1, the overall thermodynamic system includes a power supply, an alkali metal vapor cell, an electric heating plate, a temperature controller, an Ag-NWs transparent heating film, and temperature sensors. In this study, the PEEK shell serves as a thermal insulator, which prevents heat exchange between the system and its external environment. The PEEK material’s low thermal conductivity ensures minimal heat transfer, contributing to the overall thermal isolation of the system.

The power supply is used to provide the necessary 40 V DC voltage for the experiment. The DC voltage is converted into 12 V 100 kHz high-frequency AC by the heating plate, which is then input to the temperature controller and connected to the Ag-NWs’ transparent heating film to heat the vapor chamber. Temperature and its distribution within the chamber are measured using PT100 temperature sensors and an infrared thermal imaging camera. The heating device is equipped with an insulating layer to minimize heat loss and enhance thermal efficiency. The external structure is supported by PEEK, while the internal component is filled with aerogel, which has a thermal conductivity coefficient of 0.013–0.024 W/(m·K). The internal structure is illustrated in Figure 1.

**Figure 2:**
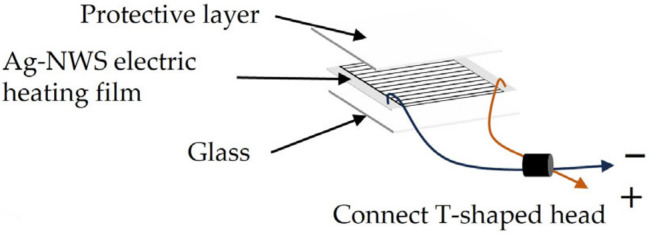
Ag-NWs’ transparent heating film structure.

The structure of the Ag-NWs’ transparent heating film is illustrated in Figure 2, with a size of 20 × 20 mm. Silver-nanowires are nanoscale elongated structures composed of silver atoms, with diameters typically ranging from 1 to 100 nanometers and lengths extending to several micrometers or more. Due to their high electrical conductivity, excellent transparency, and superior thermal conductivity, silver nanowires are advantageous for the fabrication of heating films, as they not only maintain high transparency but also exhibit excellent electrical conductivity [47,48,49]. The transparent heating film, composed of silver nanowires (Ag-NWs), is supported on a 1 mm-thick conductive glass substrate with high optical transparency. The initial stage involves the pre-treatment of the silver nanowires. The surface of the silver nanowires may contain impurities, which are removed by immersing them in an ethanol solution and employing ultrasonic cleaning for surface purification. Hydrogen gas is then utilized as a reducing agent to chemically reduce and eliminate the oxide layer. The silver nanowires are subsequently mixed with a surfactant in a solvent, and ultrasonic dispersion is applied to achieve a homogeneous distribution of the nanowires within the solution, facilitating their subsequent application. A dispersion of silver nanowires is subsequently applied via spray coating to form a 450 nm-thick Ag-NWs film on the substrate, followed by a drying process. The silver nanowire-coated glass substrate is then subjected to an annealing process in an inert gas atmosphere (e.g., helium) to prevent oxidation of the silver and to enhance its conductivity and adhesion. Patterning is subsequently performed using photolithography, followed by etching. This is followed by the deposition of a 0.6 µm-thick silicon nitride insulating layer using magnetron sputtering. Finally, a 600 nm-thick silver film was deposited via sputtering to serve as the electrode, followed by the application of a 300 nm-thick SiO_2_ layer as a protective coating. During the experimental process, adjustments were continually made to the density and thickness of the silver nanowires in the heating film to meet our heating requirements while ensuring optical transparency. Ultimately, a density of 20 mg/cm^3^ and a thickness of 450 nm were determined. The final experimental results also indicated that the optimized heating film achieved an optical transmittance of up to 90%, with a thermal response time of less than 5 s, successfully reaching the designed temperature.

## 4. Discussion

### 4.1. Simulation Comparative Analysis

The Ag-NWs’ transparent heating film, owing to its high optical transmittance, can be directly adhered to the surface of alkali metal gas chambers. In contrast, traditional flexible printed circuit (FPC) heating films lack optical transparency, necessitating the incorporation of apertures and their attachment to ovens for heat transfer via boron nitride-based systems. According to the theory of heat conduction, the temperature distribution should follow the heat conduction equation. In the simulation, we used the same equation and set the initial temperature conditions and boundary conditions. To conduct a comparative analysis of the advantages of the Ag-NWs’ transparent heating film over the FPC heating film, we performed a simulation analysis. During the simulation analysis, we maintained both heating films at an identical steady-state temperature of 120 °C and ensured that they exhibited the same thermal conductivity to guarantee the objective accuracy of the results. The thermodynamic parameters utilized in the simulation experiments are presented in Table 1.

In the simulation experiments, the dimensions of the alkali metal vapor cell were established as 20 mm × 20 mm × 20 mm. The Ag-NWs’ transparent heating film was employed for surface heating during the experiments. This method demonstrates significant advantages in heating uniformity and response speed when compared to conventional heating methods. Furthermore, it effectively minimizes interference from external factors, conserves space, and facilitates miniaturization.

**Figure 3:**
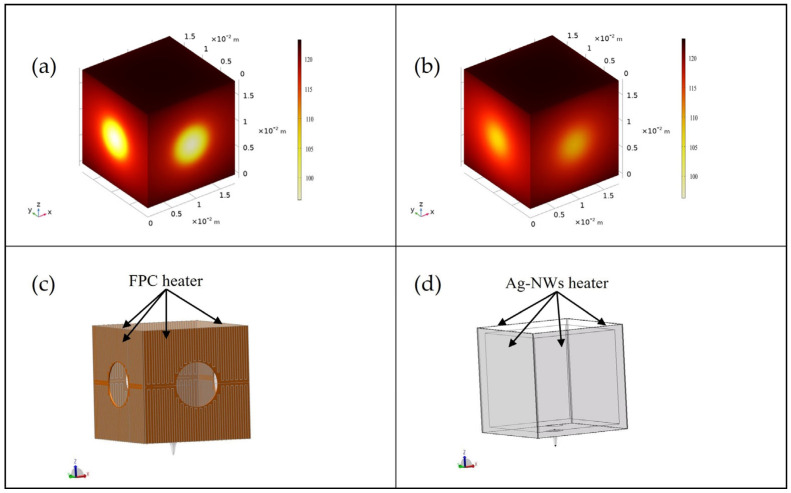
Simulation results of the interior walls and surrounding components of the alkali metal chamber. (**a**) Heating the alkali metal chamber using FPC heating film; (**b**) heating the alkali metal chamber using Ag-NWs’ transparent heating film; (**c**) FPC heating film structure; (**d**) Ag-NWs’ heating film structure.

As illustrated in Figure 3a,b, the internal temperature of the alkali metal vapor cell heated using the Ag-NWs’ transparent film is significantly higher than that of the cell heated with FPC heating film. The structures depicted in Figure 3c,d indicate that, due to the opacity of the FPC heating film, a central aperture with a diameter of 8 mm must be maintained. Consequently, the alkali metal vapor cell cannot be fully covered by the heating film, resulting in a temperature gradient during the heating process, characterized by lower temperatures in the center compared to the edges, which is notably larger than that observed with the Ag-NWs transparent film. This phenomenon leads to uneven atomic polarization within the alkali metal vapor cell, thereby adversely affecting the sensitivity of the atomic magnetometer. In contrast, the alkali metal vapor cell heated with the Ag-NWs’ transparent film exhibits a smaller temperature gradient, which effectively mitigates this issue.

### 4.2. Experiment

**Figure 4:**
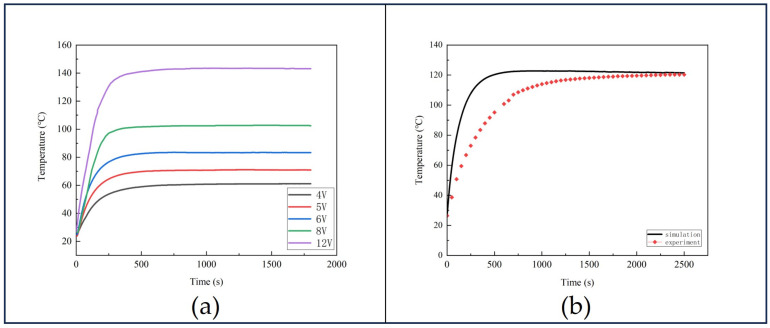
(**a**) Heating temperature of Ag-NWs’ transparent heating film at different voltages. (**b**) Simulation temperature curve (black) vs. experimental temperature curve (red).

To investigate the uniformity of the internal temperature in the alkali metal chamber heated by the Ag-NWs’ transparent heating film, we first conducted tests on the Ag-NWs’ transparent heating film itself. By applying different voltages, the heating film can achieve various temperatures, as shown in Figure 4a. In the experiment, the voltage applied to the heater is expressed as the root mean square (RMS) value, which allows us to describe the power output more accurately. The waveform used to drive the heater is a square wave, which provides stable power output. Temperature control is achieved through pulse width modulation (PWM), where the width of the pulses is adjusted to control the average power of the heater, thus enabling precise temperature control. Experimental results demonstrate that, at 4 V voltage, the Ag-NWs’ transparent heating film reaches 60 °C within 600 s and maintains a stable temperature for the next 1200 s. At 12 V, the heating temperature can reach 140 °C and remains stable thereafter. In subsequent experiments heating the alkali metal chamber, heating was conducted at 12 V, and temperature measurements were taken using a temperature sensor (PT100) and thermal imaging camera to assess internal temperatures. Figure 4b presents the measurement results obtained with the PT100 temperature sensor positioned at the upper part of the alkali metal chamber.

From Figure 4b, it can be observed that when applying 12 V to the Ag-NWs’ transparent heating film, the simulated internal temperature of the alkali metal chamber reaches 123 °C, while the actual experimental temperature is only 120.3 °C. This discrepancy is attributed to the inability of the insulation structure in this study to eliminate thermal radiation between the chamber and the surroundings, leading to heat loss from the chamber. Therefore, there exists a certain deviation between theoretical and experimental data.

**Figure 5:**
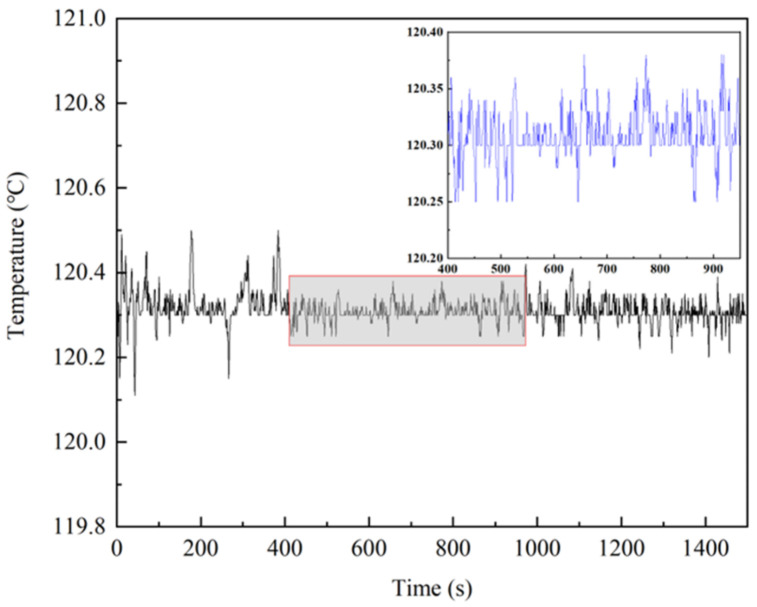
Alkali metal gas chamber temperature stability curve.

To evaluate the heating stability of the Ag-NWs’ transparent heating film gas chamber, temperature data were continuously recorded for 1500 s when the alkali metal gas chamber stabilized at approximately 120.3 °C. The resulting temperature curve of the alkali metal gas chamber is illustrated in Figure 5. As shown in the figure, the maximum and minimum temperatures of the alkali metal gas chamber are 120.5 °C and 120.1 °C, respectively, with a temperature variation of ±0.2 °C. The temperature fluctuations are minimal and can be considered negligible. When the system is in thermal equilibrium, further improvement in insulation structure can enhance the system’s stability.

**Figure 6:**
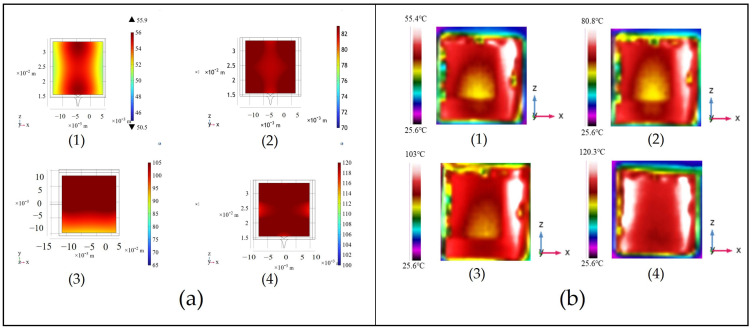
(**a**) Simulation results for 1, 2, 3, and 4 films of Ag-NWs’ transparent heating films. (**b**) Thermal maps of internal temperatures for 1, 2, 3, and 4 films of Ag-NWs’ transparent heating films.

During the simulation process, it was observed that the temperature uniformity and achievable temperature in the alkali metal chamber varied with the addition of different numbers of heating films. As shown in Figure 6(a1), when one piece of the Ag-NWs’ transparent heating film was added, the temperature reached by the alkali metal was only 60 °C, significantly lower than the operational temperature, and the internal temperature uniformity was poor. In Figure 6(a2), with the addition of two pieces of the Ag-NWs’ transparent heating film, the temperature in the alkali metal chamber noticeably increased to 80 °C, accompanied by a significant improvement in temperature uniformity. Finally, with four pieces of the Ag-NWs’ transparent heating film added, the temperature in the alkali metal chamber reached 120 °C, and the internal temperature uniformity became consistent.

To analyze the temperature distribution inside the alkali metal chamber using a thermal imaging camera, the Ag-NWs’ transparent heating film was not applied to all six faces of the chamber. Once the internal temperature of the alkali metal chamber reaches a stable state, an infrared thermal imager, capable of providing real-time thermal images at a resolution of 160 × 120 pixels, is positioned along the *Y*–axis to capture the temperature distribution within the chamber. The resulting temperature distribution is illustrated in Figure 6b.

From Figure 6b, it is evident that, when adding different numbers of heating elements, the achievable temperature within the alkali metal chamber varies significantly, and there are notable differences in internal uniformity as well. As shown in Figure 6(b1), when only one heating element is added, the alkali metal chamber reaches a maximum temperature of only 55.4 °C. This is because the heating element is only attached to one side of the chamber, resulting in poor internal temperature distribution due to heat loss, showing edges hotter than the center. Both the alkali metal chamber and the heating elements are made of quartz glass with a thermal conductivity of 1.4 W/(m·K), which hinders efficient heat transfer within the system, concentrating heat on the contact surface of the heating element. The heat inside the alkali metal chamber mainly comes from thermal radiation emitted by the chamber walls. Additionally, there is a gas handle at the bottom of the alkali metal chamber, which increases the distance for heat transfer, resulting in lower temperatures at the bottom compared to the top. Figure 6(b2) shows that, with two heating elements added, the temperature of the alkali metal chamber increases significantly, and the internal temperature uniformity is greatly improved. Ultimately, to heat the alkali metal chamber to the required operating temperature and maintain high internal temperature uniformity, it was decided to add heating elements to all four sides of the chamber. The experimental results in Figure 6(b4) demonstrate that when four heating elements are added to the surfaces of the alkali metal chamber, the chamber temperature reaches 120.3 °C, with a much more uniform internal temperature distribution.

In addition to the previously mentioned issue of uneven temperature distribution, a significant factor affecting the performance of atomic magnetometers is the inherent drawback of the electric heating method, specifically the magnetic field noise generated during the heating process. To further investigate the impact of magnetic noise produced during the heating process of the Ag-NWs’ transparent heating films, a series of experiments were designed. Initially, the heating element was connected to a power supply, with the magnetometer positioned in close proximity to ensure accurate detection of the magnetic field. Subsequently, a temperature sensor was affixed to the surface of the alkali metal vapor cell to monitor the heating temperature in real time, ensuring that the heating film achieved and maintained the required experimental temperature. In electromagnetism, the magnetic field strength generated by a material under a specific current is typically expressed in terms of magnetic permeability, measured in nT/mA (nanotesla per milliampere). A direct current voltage was applied to the heating film, and measurements were conducted using the commercial CTM-6W magnetometer. Under varying temperature conditions, the maximum recorded value was 0.1454 nT/mA, with an average value obtained from multiple measurements being 0.1143 nT/mA. When the alkali metal vapor cell was heated simultaneously from all four sides using the Ag-NWs’ transparent heating film, the heating films were connected in parallel. When the required operating temperature is reached, the measured magnetic field noise ranges from 4.16 nT to 7.27 nT, with an average value of 5.715 nT. Under identical heating conditions, experimental results indicated that the magnetic field noise produced was superior to that of the FPC heating film. The current supplied to the Ag-NWs’ transparent heating film was sourced from a power amplifier integrated within the heating circuit. This amplifier typically operates at a noise level of 7 dB, rendering the generated noise negligible. Furthermore, the heating circuit utilizes high-frequency alternating current, which, in conjunction with the structure of the heating film, can further mitigate magnetic noise interference from the heating film.

**Figure 7:**
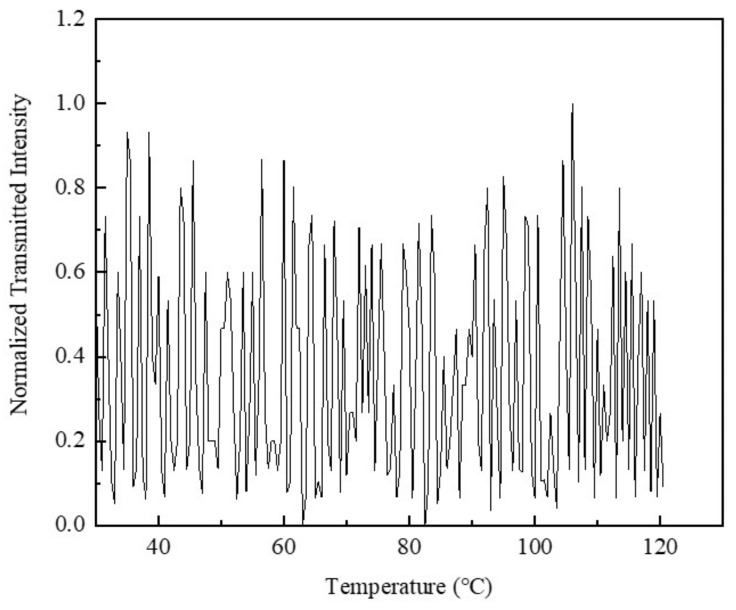
Curve of normalized transmitted intensity as a function of temperature.

An experimental design was employed to evaluate the optical and electrical properties of Ag-NWs’ transparent heating films. Circularly polarized light was utilized to assess their transmittance, while linearly polarized light was used to examine their impact on polarization states. Initially, a photometer was used to measure the power without the heating film, and this measurement was compared to the power obtained with the heating film in place. Consequently, the transmittance of the single-layer Ag-NWs’ transparent heating film was determined to be 89.31% ± 1.01%. The polarization state is a critical parameter in assessing the performance of the heating film. Since the Ag-NWs’ transparent heating film can be directly adhered to the surface of an alkali metal vapor chamber, it is essential that the polarization state of the incident light remains strictly consistent with that of the transmitted light. When the heating film is heated to 120 °C, the transmitted light intensity is 88.67% ± 0.53% of the intensity when it is unheated. As illustrated in Figure 7, we normalize the transmitted light intensity to eliminate the fluctuations in light source power and the effects of other experimental conditions, making the results more comparable under different experimental setups. It was observed that the light intensity remained relatively unchanged across different temperatures, indicating that the Ag-NWs’ transparent heating film maintains optical uniformity and does not alter the polarization state of the light under varying thermal conditions. This implies that the Ag-NWs’ transparent heating film does not exhibit selective absorption or scattering of light with different polarization states. The extinction ratio is also a measure of polarization stability. A lower extinction ratio indicates a higher degree of polarization in the polarized light produced by the optical device. The Ag-NWs’ transparent heating film was placed within the optical path for comparative analysis. The extinction ratio was measured under three distinct modes. By rotating the analyzer, it was observed that the power detected by the photometer was maximized when the analyzer’s principal axis was aligned with the primary polarization direction of the incident laser. Conversely, the power detected was minimized when the analyzer’s axis was orthogonal to the primary polarization direction. The extinction ratio can thus be calculated using the following formula:

(5) $$PER=10\log_{10}\frac{P_{\max}}{P_{\min}}(dB).$$

where, $P_{\max}$ represents the maximum power of the signal, $P_{\min}$ represents the minimum power of the signal, dB is the logarithmic unit of power ratio used to express the extinction ratio. First, the extinction ratio was measured without the addition of the Ag-NWs’ transparent heating film, yielding a calculated value of 14.42 dB. Subsequently, placing the Ag-NWs’ transparent heating film in the optical path and rotating the analyzer resulted in an extinction ratio of 13.96 dB. Comparing these two values, the extinction ratio with the Ag-NWs’ transparent heating film added was 96.8% of the value obtained without it. After applying electric heating to the Ag-NWs’ transparent heating film and repeating the experiment with it in the optical path, the extinction ratio was measured at 13.89 dB. This corresponds to 96.3% of the original extinction ratio, indicating a decrease of 0.5% compared to the non-heated state, which is negligible. Therefore, this demonstrates that the Ag-NWs’ transparent heating film can be directly applied to the surface of alkali metal chambers for heating while maintaining high transmittance and without altering the polarization state.

## 5. Conclusions

This paper presents a non-magnetic transparent heating film based on Ag-NWs, suitable for scalable production on a single wafer. By simulating and optimizing the parameters of the Ag-NWs’ thin film, heating uniformity is enhanced. Experimental results indicate that the film can withstand temperatures of up to 140 °C, with an average magnetic flux coefficient of 0.1143 nT/mA across various operational modes. A comparison with traditional Flexible Printed Circuit (FPC) heating films shows that, under 100 kHz AC heating currents, the Ag-NWs’ non-magnetic transparent heating film provides a more uniform temperature field than FPC heating films. The Ag-NWs-based heating film designed in this study can be applied not only in atomic magnetometers but also in other chip-level atomic sensors such as chip-level atomic clocks and gyroscopes. Future enhancements in the Ag-NWs’ fabrication process hold potential for further improving the heating film’s temperature control and magnetic field shielding capabilities.

## Figures and Tables

**Table 1:** Simulation material parameters.

Material (Component)	Parameter	Value
Helium (Gas)	Specific heat rate	1.66
Borosilicate (Glass)	Constant press heat capacity	850 J/(kg·K)
	Density	2400 kg/m^3^
	Thermal conductivity	1.1 W/(m·K)
Boron Nitride (Oven)	Thermal conductivity	25.1 W/(m·K)
Ag-NWs (Heating Film)	Thermal conductivity	0.93 W/(m·K)

## Data Availability

Data is contained within the article.
